# The “Petechiae in children” (PiC) study: evaluating potential clinical decision rules for the management of feverish children with non-blanching rashes, including the role of point of care testing for Procalcitonin & *Neisseria meningitidis* DNA – a study protocol

**DOI:** 10.1186/s12887-018-1220-x

**Published:** 2018-07-30

**Authors:** Thomas Waterfield, Mark D. Lyttle, Derek Fairley, James Mckenna, Kerry Woolfall, Fiona Lynn, Julie-Ann Maney, Damian Roland, Aoife Weir, Michael D. Shields, on behalf of Paediatric Emergency Research in the UK and Ireland (PERUKI)

**Affiliations:** 1 Centre for Experimental Medicine, Wellcome Wolfson Institute of Experimental Medicine, Queen’s University Belfast, Belfast, UK; 2 Belfast Health and Social Care Trust, Belfast, Northern Ireland; 3 Bristol Royal Hospital for Children, Upper Maudlin Street, Bristol, UK; 4 Faculty of Health and Applied Sciences, University of the West of England, Bristol, UK; 5 Psychological Sciences, University of Liverpool, Liverpool, UK; 6 School of Nursing and Midwifery Centre for Evidence and Social Innovation Queen’s University Belfast, Belfast, UK; 7 SAPPHIRE Group, College of Life Sciences, University of Leicester and Paediatric Emergency Medicine Leicester Academic (PEMLA) Group, Leicester, UK

**Keywords:** Meningococcal, Meningitis, Sepsis, Management, Loop-mediated-isothermal AMPlification, Procalcitonin

## Abstract

**Background:**

Children commonly present to Emergency Departments (ED) with a non-blanching rash in the context of a feverish illness. While most have a self-limiting viral illness, this combination of features potentially represents invasive serious bacterial infection, including meningococcal septicaemia. A paucity of definitive diagnostic testing creates diagnostic uncertainty for clinicians; a safe approach mandates children without invasive disease are often admitted and treated with broad-spectrum antibiotics. Conversely, a cohort of children still experience significant mortality and morbidity due to late diagnosis. Current management is based on evidence which predates (i) the introduction of meningococcal B and C vaccines and (ii) availability of point of care testing (POCT) for procalcitonin (PCT) and *Neisseria meningitidis* DNA.

**Methods:**

This PiC study is a prospective diagnostic accuracy study evaluating (i) rapid POCT for PCT and *N. meningitidis* DNA and (ii) performance of existing clinical practice guidelines (CPG) for feverish children with non-blanching rash. All children presenting to the ED with a history of fever and non-blanching rash are eligible. Children are managed as normal, with detailed prospective collection of data pertinent to CPGs, and a throat swab and blood used for rapid POCT. The study is running over 2 years and aims to recruit 300 children.

Primary objective:

- Report on the diagnostic accuracy of POCT for (i) *N. meningitidis* DNA and (ii) PCT in the diagnosis of early MD
- Report on the diagnostic accuracy of POCT for *PCT* in the diagnosis of Invasive bacterial infection

Secondary objectives:

- Evaluate the performance accuracy of existing CPGs
- Evaluate cost-effectiveness of available diagnostic testing strategies
- Explore views of (i) families and (ii) clinicians on research without prior consent using qualitative methodology
- Report on the aetiology of NBRs in children with a feverish illness

**Discussion:**

The PiC study will provide important information for policy makers regarding the value of POCT and on the utility and cost of emerging diagnostic strategies. The study will also identify which elements of existing CPGs may merit inclusion in any future study to derive clinical decision rules for this population.

**Trial registration:**

NCT03378258. Retrospectively registered on December 19, 2017.

## Background

Early diagnosis of meningococcal disease (MD) is associated with improved outcomes including reduced morbidity and mortality [1, 2]. However during its prodrome phase invasive MD, which most often presents with a fever and non-blanching rash (NBR), is indistinguishable from many self-limiting viral infections, creating a significant diagnostic challenge for clinicians. This combination of features is a common presentation to Emergency Departments (ED) and inevitably leads to caution amongst clinicians, resulting in admission and administration of broad spectrum antibiotics to large numbers of children who do not have MD. Despite this cautious approach a cohort of children are still diagnosed late [1, 3]. Paediatric Emergency Research in the UK and Ireland (PERUKI, a research collaborative) highlighted these challenges in the context of a paucity of relevant evidence, and identified the derivation of a clinical decision rule (CDR) for the management of feverish children with NBRs as a priority for future research [4, 5].

## Current UK guidance

There are currently two clinical practice guidelines (CPG) in widespread use in the UK for the management of children with NBR. These are:

- National Institute for Health and Care Excellence (NICE) CG102 “Meningitis (bacterial) and meningococcal septicaemia in under 16s: recognition, diagnosis and management”
- The Newcastle-Birmingham-Liverpool algorithm [3, 6].

These CPGs were developed based on data collected prior to the introduction of Meningococcal vaccines into the UK vaccination schedule (Table 1) [6–9]. Both CPGs advocate a similar and cautious approach to management of non-blanching rashes in children. Both CPGs are reported to be highly sensitive for the diagnosis of MD (NICE 97%) and (NBL) 100% [6]. The specificity of the two CPGs has been estimated as 50% (NICE) 82% (NBL) [6]. The most significant difference between the two CPGs is that in the NBL CPG does not include fever or history of fever whereas the NICE CPG requires a fever (or history of fever) and NBR [6]. Data on sensitivity and specificity of the existing CPGs was collected largely before the introduction of meningococcal B and C vaccination meaning their performance in the current post vaccination era is unknown.

**Table 1:** Current UK vaccination against invasive meningococcal disease

Current UK Vaccination Against Invasive Meningococcal Disease		
Vaccine (Serogroups)	Year Introduced into schedule	Age given
B	2015	8 weeks, 16 weeks, 1 year
C	1999	1 year^a^
ACWY	2015	14 years, University students 19–25 years

^a^Prior to 2016 meningococcal C vaccine was also administered at 12 weeks

### Point of care testing

Current guidance suggests a range of investigations aimed at establishing the risk of invasive disease, and/or identifying a pathogen. To date, these have predominantly been performed in laboratory settings. However recent advances in technology have created the potential for point of care testing to be employed, either to identify MD, or to stratify risk of invasive disease. The two which offer most promise in the context of NBR are Loop Mediated Isothermal Amplification for Meningococcal DNA (LAMP MD) and procalcitonin (PCT).

#### Lamp md

**Fig. 1:**
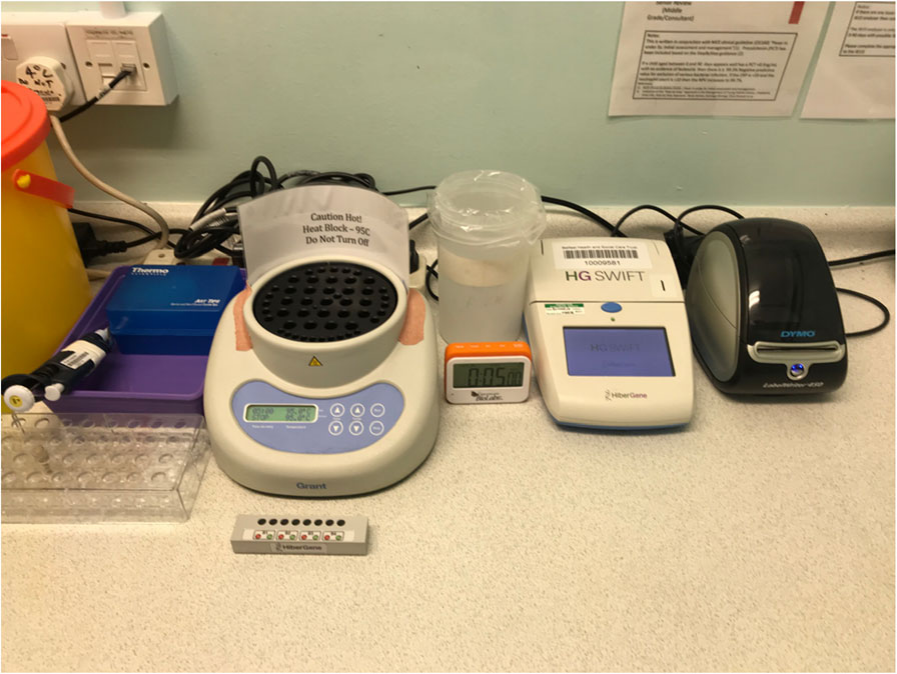
Hibergene LAMP-MD testing equipment

LAMP-MD is a rapid molecular amplification test for the detection of all serogroups of *N. meningitidis* DNA. It can be performed in the ED on a bench top analyser and provides results within 30 min (Fig. 1). Initial testing has shown that LAMP-MD has superior sensitivity (0.89 [95%CI 0.72–0.96]) and specificity (1.0 [95%CI 0.97–1.0]) in diagnosing MD than traditional tests (C-reactive protein [CRP] and White Blood Cell counts [WBC]) recommended by NICE [10, 11]. The LAMP-MD test is now available commercially and is CE-IVD approved. The test performs equally well on blood samples and throat swabs, [10] with results available more quickly from throat swabs due to a shorter and simpler DNA extraction process.

#### Procalcitonin

**Fig. 2:**
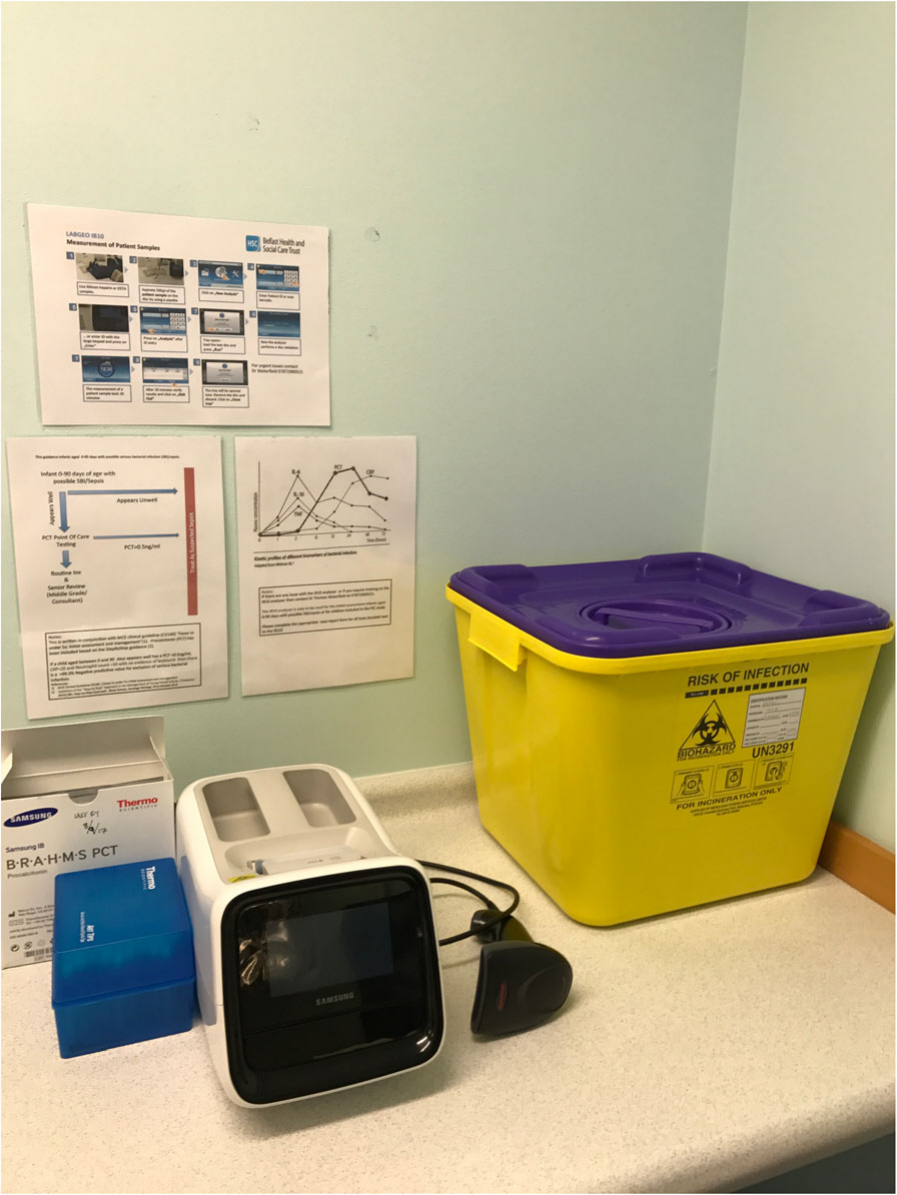
Samsung IB10 (BRAHMS) Procalcitonin

Procalcitonin (PCT) is the precursor for calcitonin and is produced by parafollicular cells. It is a 116-amino acid protein that has roles in calcium metabolism. PCT is elevated during infection and typically rises within 2 h of the onset of a bacterial infection. A recently published meta-analysis of 6 studies, including 881 children, found PCT to be more sensitive (0.89 [95%CI 0.76–0.96]) and specific (0.74 [95%CI 0.4–0.92]) in diagnosing early MD than CRP or WBC [12]. A further strength of PCT is that it rises within 2 h of onset of bacterial illness and peaks at around 6 h (much earlier than CRP) [12]. ED clinical staff can perform PCT POCT on a bench top analyser with results available within 20 min. (Fig. 2)

## Methods/design

As a diagnostic accuracy study our Petechiae in Children (PiC) study adheres to the STARD criteria for the reporting of diagnostic accuracy studies [13]. The gold standard test against which our outcomes are measured is quantitative PCR (qPCR) for *N. meningitidis* DNA in a sterile body site (blood or CSF). Or confirmation of an invasive bacterial infection through positive culture or qPCR of a bacterial pathogen.

### Study registration

The PiC study was registered at https://www.clinicaltrials.gov (trial registration: NCT03378258) on the 19th of December 2017. At the time of registration 24 patients had been recruited to the PiC study which opened on the 22nd of November 2017.

### Objectives

The co-primary outcomes are the diagnostic accuracy (sensitivity, specificity, positive predictive value, and negative predictive value) of:

- POCT for (i) *N. meningitidis* DNA and (ii) PCT in the diagnosis of MD in children with fever and NBR
- POCT for *PCT* in the diagnosis of Invasive bacterial infection

Secondary objectives:

- Evaluate the performance accuracy of existing CPGs in identifying MD
- Evaluate cost-effectiveness of available diagnostic testing strategies
- Explore views of (i) families and (ii) clinicians on research without prior consent (RWPC) using qualitative methodology
- Report on the aetiology of NBRs in children with a feverish illness

### Study population and setting

#### Inclusion criteria

- All children < 14 years of age attending the ED with reported or recorded fever (≥38 °C) and NBR.
- Unwell appearing children with features of meningococcal sepsis/meningitis as outlined in the National Institute for Health and Care Excellence (NICE) CG102 “Meningitis (bacterial) and meningococcal septicaemia in under 16s: recognition, diagnosis and management” [3].

#### Exclusion criteria

1. Children with pre-existing haematological conditions such as haematological malignancy, idiopathic thrombocytopenic purpura (ITP) and coagulopathy will be excluded.
2. Existing Henoch-Schonlein purpura (HSP) under follow up

### Sample size justification

We have calculated that we need 203 test negative patients (negative LAMP & low procalcitonin) to estimate a negative predictive value (NPV) of 95% or greater with confidence intervals of +/− 3% (Calculation below). Disease prevalence is estimated at 15% or lower, based on preparatory work in our centre and other epidemiologic studies, and we anticipate a combined refusal of consent and dropout rate of 10%. We therefore aim to recruit a total of 300 patients to achieve this. We have chosen to focus on NPV because with possible MD the emphasis is on exclusion of a life-threatening infection and as such and test or CPG must have a high NPV.

### NPV Calculation

Nnegative = Z_0.025_^2^x (NPV(1 ‐ NPV))/W^2 ^ = 1.96^2^x (0.95(1 ‐ 0.95))/0.03^2^ = 203

### Assessments and procedures

**Fig. 3:**
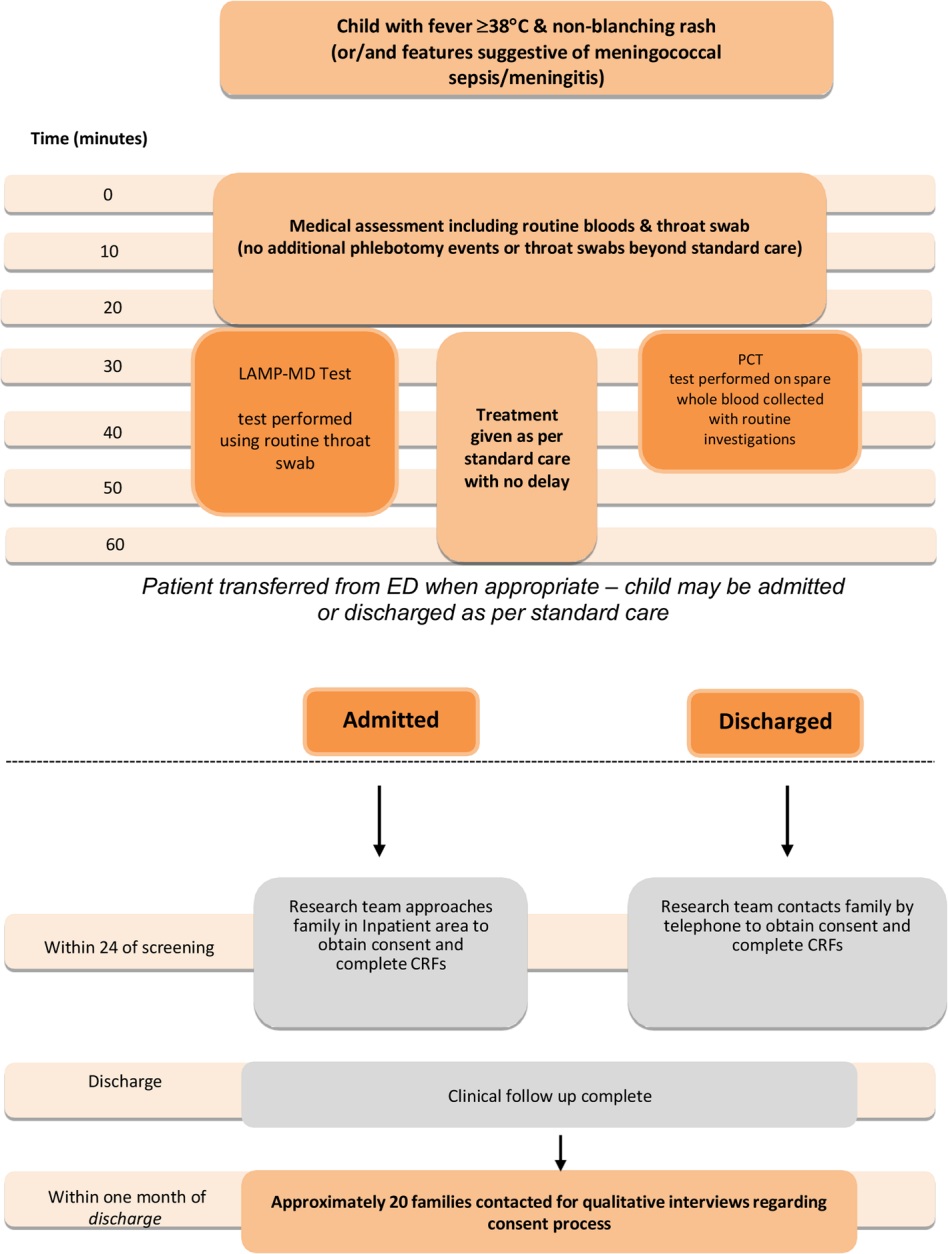
Flow diagram for study proceduress

The required assessments and procedures are outlined in Table 2. Eligible children undergo POCT in parallel to their standard ED care with no delay (Fig. 3). Residual specimens beyond what is needed for standard care is tested using the Hibergene LAMP-MD and the Samsung IB10 (BRAHMS) PCT.

**Table 2:** PiC Study assessments

	In ED	Follow-up		
		4 h	0–24 h	Within 1 Month
Consent discussion	X	X	X	
Assessment of eligibility criteria	X			
POCT	X			
Laboratory assessments – routine bloods and throat swabs	X			
Notes review and CRF completion by member of research team			X	
Review of medical history by member of research team			X	
Qualitative interview conducted by TW				X

### Performing index tests - blood samples & throat swabs

POCT is performed by ED clinical staff, with bespoke training provided by the research team, and a training log maintained. Members of the research team are contactable to provide support as required. Index tests are performed as soon as possible, and are done prior to result of the reference standard test (Quantitative PCR) being available.

#### LAMP-MD

A throat swab taken as part of routine care is mixed in a sample buffer; a small aliquot of this (50 μl) is used, and the main sample is forwarded to the laboratory as per normal practice. Standard testing includes molecular testing for *N. meningitidis*, human enteroviruses, and a series of other viral targets using reference-laboratory real-time PCR methods. The aliquot drawn in the ED is analysed using the Hibergene LAMP-MD POCT test (giving positive/valid, negative/valid or invalid result) – Fig. 1.

#### Procalcitonin

0.5 ml of blood is taken from the samples collected as part of routine care. POCT PCT testing is done on this sample in the ED using the Samsung IB-10 analyser with a positive threshold level of > 1.93 ng/ml. If insufficient blood is obtained, then routine testing is prioritised. Routine blood testing includes WBC, CRP, coagulation screen, blood gas, and molecular testing for *N. meningitidis*, human enteroviruses, and other viral and bacterial targets (including *Streptococcus pneumoniae* and *Haemophilus influenzae*) using reference-laboratory real-time PCR methods – Fig. 2.

### Reference standards

In all cases of possible MD, blood and/or CSF is tested for *N. meningitidis* DNA using quantitative crtA TaqMan PCR using standard UK laboratory methods. The reference standard test is performed by staff blinded to the result of the index test. A positive reference standard test will be used to give a diagnosis of “confirmed MD”.

In cases where the diagnosis is unclear or where the child has been diagnosed as “probable MD” by the clinical team but where the reference standard test is negative a committee of clinicians will review the anonymised medical records blinded to results of POCT and decide if the case can be given the diagnosis of “probable MD”.

For other invasive bacterial infections, the reference standard test is positive culture or qPCR of bacterial pathogen from a sterile body site (blood/CSF) performed by staff blinded to the result of the index test.

### Case report form (CRF)

All children recruited to the study will have a standardised CRF completed by a member of the research team. Regular meetings will be arranged to ensure standardisation of data collection. Demographic data will be collected as will data pertinent to current CPGs. This includes, but is not limited to, vital signs, overall wellness, duration of illness, appearance and distribution of the rash, any spread of the rash over time (including the first 4 h in hospital). Data will be collected on investigations performed, treatments given, final diagnosis, length of stay and survival to discharge. Where data is unclear from the clinical record the researcher will collect additional information from the child’s parent and attending physician.

### Interviews with parents and clinicians

The study includes interviews with (*n* = ~ 20) parents and (*n* = ~ 5–10) clinicians involved with recruitment and consent processes to explore their views on RWPC in this study.

During the consent process, parents are asked whether they consent to a qualitative telephone interview, which takes place within one month of their child’s discharge from hospital. All parents are invited to consent for a qualitative interview, including those who decline the use of their child’s information in the study. Interviews will be conducted until data saturation is reached [14].

All interviews with parents and clinicians will be conducted by TW. Any distress during the interviews will be managed with care and compassion and participants will be free to decline to answer any questions that they do not wish to answer or to stop the interviews at any point. Consent for audio recording will be sought. If consent is not provided then the interview will not continue.

### Research without prior consent (deferred consent)

Informed consent is a process initiated prior to an individual agreeing to participate in a study and continues throughout the individual’s participation. When consent is deferred, an individual is agreeing to the use of data that had already been collected for study purposes and for continued participation in the study [15, 16]. Research without prior consent in children has been shown to be appropriate and well accepted by parents when conducted in emergency situations and when information and opportunities for consent are offered at an appropriate time [15, 16]. In the PiC study we intend to assess the performance of rapid bedside tests in the diagnosis of a life-threatening emergency. In this situation, every minute counts and it is therefore not possible or appropriate to delay testing whilst consent is obtained (even for a few minutes). Following testing parents will be approached at the earliest appropriate opportunity and ideally within 24 h.

#### Approaching parents

A member of the research team will be notified of the participation of the child in the study and will approach the parent to seek consent as soon as possible after undergoing POCT (ideally within 24 h). In the majority of cases this will take place on a ward or in the ED. Consent will only be sought once the child is stable and following consultation with the clinical team caring for the child in line with best practice recommendations [15–17].

#### Approaching parents in the ED

Not all children with a fever and NBR are admitted. If the child appears well the clinician may choose to perform investigations and observe the child in the ED. Following a period of observation (typically 4–6 h) the child may be discharged if they appear well and testing is reassuring. In this group, we intend to seek consent prior to discharge. Before approaching the family in the ED, the researcher checks with clinical staff that the child is stable and timing is appropriate. An ED clinician explains the nature of the study to the parent and invites them to discuss the study with the researcher.

Some children will be discharged before consent can be obtained. In this instance, an ED clinician will contact the parent by telephone (maximum of 3 attempts) to explain the study and invite parents to discuss the study with a researcher, who then explains the reasons for RWPC, and how to opt in or out of the study. The parent is sent a patient information sheet (PIS), consent form and follow up letter. This letter explains the study, reasons for RWPC, how to opt in or out of the study, and provide contact details for the research team. If after 4 weeks there is no response, a follow up letter, PIS, and consent form are sent to the family. This explains the study, reasons for RWPC, how to opt in or out, and provides contact details for the research team. This letter also confirms that if no consent form is received within 4 weeks then the participant’s data will not be included in the study.

#### Approaching parents on the wards

The research team is notified of enrolment and approaches the parent to seek consent as soon as possible after undergoing POCT (ideally within 24 h). Based on CONNECT best practice guidance for performing RWPC the researcher checks with the clinical team that the participant is stable and that timing is appropriate before approaching the parent on the ward [16]. If the participant’s condition has not stabilized additional time will be allowed [16]. A member of the ward team explains the nature of the study and invites the parent to talk with the researcher.

#### Death prior to consent being sought

This will be rare, but almost certainly will occur. When a participant dies before consent has been sought TW will obtain information from colleagues and establish the most appropriate practitioner to notify parents of the research involvement.

Consent can be sought from parents following the death of their child and prior to the parent’s departure from the hospital. However, it is at the discretion of the clinical staff to determine if this is appropriate for each individual family. It maybe that it is not appropriate for consent to be obtained prior to discharge [15, 16].

Following the death of a child at the RBHSC it is routine practice to invite the parents to a meeting with the consultant in charge of their child’s care. This usually takes place 4–6 week after death. At this meeting, the consultant will be asked to explain the PiC study, reasons for RWPC, how to opt in or out, and provide contact details for the research team.

Following the meeting, 4 weeks is allowed for the family to contact the research team. If no contact is made then a personalised letter including the PIS and consent form is sent to the family. This explains the study, reasons for RWPC, how to opt in or out, and provides contact details for the research team. If after 4 weeks after sending the initial letter to the bereaved family, there is no response, a follow up letter along with the parent representative information sheet and consent form will be sent to the bereaved family. This second letter will explain the study, reasons for research without prior consent (deferred consent), how to opt in or out of the study and provide contact details if parents wish to discuss the study with a member of the research team (either in person or by telephone). In addition, this letter will also confirm that if no consent form is received within 4 weeks of the letter being sent then the participant’s data will NOT be included in the study.

#### Deferred consent declined/not obtained

If RWPC is declined or not obtained the child’s data will not be included. TW will maintain a record of all instances of declined/not obtained consent.

#### Withdrawal of consent

Consent may be withdrawn at any time without providing a reason and without being subject to any resulting detriment. The rights and welfare of the patients will be protected and the quality of medical care will not be adversely affected if they decline to participate in the study. TW will maintain a record of all those that withdraw consent to participate in the study.

*Research Ethics Committee (REC) and Institutional Review Board (IRB) opinion*

*The Northern Ireland REC and the Belfast Trust IRB have both reviewed the PiC protocol and provided a favorable outcome including the use of research without prior consent (deferred consent) as described above (Project ID 224660).*

### Statistical analysis

In keeping with the objectives we will report:

Primary:

1. The diagnostic accuracy of POCT for (i) *N. meningitidis* DNA and (ii) PCT in the diagnosis of early MD against the reference standard (qPCR) for *N. meningitidis* DNA in blood/CSF). The sensitivity, specificity, NPV and PPV will be reported.
2. The diagnostic accuracy of POCT for *PCT* in the diagnosis of early invasive bacterial infection against the reference standard of positive culture from a sterile site (blood/CSF). The sensitivity, specificity, NPV and PPV will be reported.

Secondary:

1. The diagnostic accuracy of existing UK guidance in the diagnosis of early MD against the reference standard qPCR) of blood/CSF. The sensitivity, specificity, NPV and PPV will be reported. McNemar’s test will be performed to determine the significance in performance of different guidance. Where possible the effect of incorporating POCT into existing guidance will be explored and reported in term of sensitivity, specificity, NPV and PPV.
2. Report on the aetiology of NBRs in children with a feverish illness
3. The epidemiology of the population of children who present with fever and a NBR

### Qualitative analysis

Qualitative interview data will be transcribed verbatim, checked and anonymised as the study progresses. QSR NVivo software will be used to assist in the organization and indexing of qualitative data. Data will be analyzed thematically, informed by the constant comparison approach of grounded theory [18]. The focus will be modified to fit with the criterion of catalytic validity, whereby findings should be relevant to future research and practice.

### Cost analysis

The PiC study will include a review of the economic impact of the current management of children with fever and a non-blanching rash and to assess the possible cost analysis of the rapid POCT diagnostic tests compared to standard care from the NHS perspective. Resource use for PCT, LAMP-MD POCT and current standard tests will be collected along with unit costs to identify mean costs in delivering these tests. Resource use will be derived at the individual patient level. Clinical staff will record the time and activities undertaken for specimen collection. Associated costs will be calculated using a standard micro costing (bottom-up) approach, and will be based on clinical staff salaries plus on-costs (employer’s national insurance and superannuation contributions) and appropriate capital, administration, laboratory and training costs. These data will also inform the costs of designing and running a large multicentre study.

## Discussion

The PiC study represents a pragmatic approach to a difficult but important research question. PiC aims to determine (i) the value of novel POCT strategies, (ii) the performance of existing guidance, (iii) the role of both these elements in the derivation of a CDR to aid clinicians in this area of diagnostic dilemma, and (iv) the feasibility of deriving and validating such a CDR. We will also provide important aetiological and epidemiological data on childhood NBRs in the post-vaccine era.

### Potential risks

Clinical risks are minimal as the PiC study doesn’t involve any change to routine care. All children will still be managed according to the existing meningococcal treatment pathway in operation at the RBHSC. The results of the POCT will not be used to remove a child from the care pathway. No additional blood samples or throat swabs are required, as we use samples taken as part of standard care.

### Potential benefits

For children admitted to hospital for IV antibiotics, there is no likelihood of personal benefit from participating in this study. However for enrolled children who would otherwise be discharged directly from the ED there is potential benefit, as the results of the LAMP-MD and PCT will be made available to the clinical team. Being enrolled in this study may therefore result in the child receiving lifesaving treatment that they would have otherwise not received, with guidance provided for clinicians on meaningful test cut-points. [10, 12].

### Potential bias

As the clinical team are not blinded to the results of the index tests, there is an increased risk of bias in treatment. It was deemed ethically unacceptable to withhold these results from clinicians. However as our performance accuracy is primarily assessed laboratory tests we expect any bias to be minimized, with a residual risk remaining for cases which require a clinical decision as to the likelihood of MD in the context of negative laboratory tests. When this occurs bias will be minimized by ensuring the treating clinician is not part of this panel, with information taken solely from clinical notes. In addition the laboratory team performing reference tests will be blinded to index tests. This includes blood culture testing and qPCR for other invasive bacterial infections.

### Limitations of the study

Whilst the PiC study is powered to provide useful data on the diagnostic accuracy of POCT for PCT and *N. meningitidis* DNA and to report on the performance of existing guidance it is underpowered to define a new clinical decision rule outright.

The learning from PiC s likely to inform the design of a larger multicentre study and may also point towards areas for further guideline/clinical decision rule development.

### Study committees

#### Public & Patient Involvement Advisory Group (PPI)

The hope is that the PPI advisory group would play a full part in all aspects of the study. In particular there is a clear benefit of their involvement in the application for ethical approval and with developing resources for parents and children including the final publication of results and the development of patient information.

The chairperson of the PPI advisory group will be involved with publication writing and will be named as a co-author on the study and study protocol and all members of the PPI advisory group would be encouraged to attend the free HSCNI “Building Research Partnerships” course. Additional funding will be made available to provide training for members of the PPI advisory group so that they can confidently contribute to the study.

#### Independent study monitoring group

An independent study monitoring group chaired will oversee the quality of the research and manage any potential conflicts of interest.

## Abbreviations

CE-IVD: Conformite Europene In Vitro Diagnostics
CI: Confidence Interval
COAG: Coagulation Studies
CRF: Case Report Form
CRP: C-Reactive Protein
ED: Emergency Department
FBC: Full Blood Count
IRB: Institutional Review Board
ITP: Idiopathic Thrombocytopenic Purpura
LAMP-MD: Loop-mediated isothermal amplification – Meningococcal Disease
MD: Meningococcal Disease
NBL: Newcastle-Birmingham-Liverpool
NBR: Non-Blanching Rash
NICE: The National Institute for Health and Care Excellence
NPV: Negative Predictive Value
PCR: Polymerase Chain Reaction
PCT: Procalcitonin
PERUKI: Paediatric Emergency Research in the UK and Ireland
PiC: Petechiae In Children
PICU: Paediatric Intensive Care Unit
POCT: Point of Care Testing
PPI: Patient Public Involvement
PPV: Positive Predictive Value
QPRC: Quantitative Polymerase Chain Reaction
RBHSC: Royal Belfast Hospital for Sick Children
RCEM: Royal College of Emergency Medicine
REC: Research Ethics Committee
U&E: Urea and Electrolytes
WBC: White Blood Cells
